# Recent Advances in Antimicrobial Coatings and Material Modification Strategies for Preventing Urinary Catheter-Associated Complications

**DOI:** 10.3390/biomedicines10102580

**Published:** 2022-10-14

**Authors:** S. P. Yamini Kanti, Ildikó Csóka, Orsolya Jójárt-Laczkovich, Lívia Adalbert

**Affiliations:** Institute of Pharmaceutical Technology and Regulatory Affairs, University of Szeged, Eötvös u. 6, H-6720 Szeged, Hungary

**Keywords:** CAUTI, prevention, urinary catheter, antimicrobial coatings, regulatory science, patient management

## Abstract

In recent years, we have witnessed prominent improvements in urinary catheter coatings to tackle the commonly occurring catheter-associated urinary tract infection (CAUTI) in catheterized patients. CAUTIs are claimed to be one of the most frequent nosocomial infections that can lead to various complications, from catheter encrustation to severe septicaemia and pyelonephritis. Besides general prevention hygienic strategies, antimicrobial-coated urinary catheters show great potential in the prevention of urinary catheter-associated complications. The aim of this review is to present and evaluate recent updates on the development of antimicrobial urinary catheters in the context of the aetiology of urinary malfunction. Subsequently, we shed some light on future perspectives of utilizing 3D printing and the surrounding regulatory directions.

## 1. Introduction

The word catheter comes from the ancient Greek word “Kathienai”, which means “to send down”. A urinary catheter is a long, hollow, partially flexible tube which collects urine from the bladder and leads it to the drainage bag. Urinary catheters are available in various sizes and types and are manufactured from different polymers, mainly using silicone and latex rubber, polyvinyl chloride (PVC), and polyurethane. Foley’s catheters are the most commonly used catheters in the world, which were designed by an American urologist named Frederic Foley following the original depiction in 1929.

Indications for catheter use are outlined in several clinical scenarios, such as management of urinary retention with or without bladder outlet obstruction, management of immobilized patients (e.g., pelvic fracture), hourly urine output measurements in critically ill patients, and improved patient comfort for end-of-life care [1].

The catheterization procedure can also be performed due to urethral injury; birth defects affecting the urinary tract, kidneys, ureters or bladder stones; nerve damage; bladder weakness; tumours in the urinary tract or reproductive organs; enlarged prostate in males; and blockage of the urethra. A urinary catheter can be used in the short-term or long-term, depending upon the condition of the patient. Insertion of an indwelling urethral catheter (IDC) is an invasive procedure that should only be carried out using an aseptic technique by a nurse or doctor [2].

According to the literature, each clinic follows standard protocols for catheterization procedures with the healthcare professional’s engagement, which includes, “training, following hygiene protocols such as handwashing, use of sterile gloves, intermittent catheterization, no-touch insertion methods, assessment of urinary cultures and use of appropriate catheter materials” [3].

However, despite the mandatory hygienic procedures, catheter-associated urinary tract infections (CAUTIs) are highlighted as the most common healthcare-associated infection. [4] The definition of a CAUTI is a “urinary tract infection where an indwelling catheter was in place for more than 48 h”. According to European studies, “15–25% of hospitalized patients and 5% of patients in elderly homes have a urinary catheter and the annual costs for CAUTI accounts for £99 million every year in the United Kingdom” [5]. Therefore, it is paramount to identify critical elements in CAUTI management to prevent and tackle unnecessary complications which account for significant morbidity and mortality associated with CAUTI [6]. In this review, we aim to focus on the factors that play a key role in urinary catheter-associated complications and explore the potential of currently available catheter developments on antimicrobial coatings and material modifications in the context of urinary malfunction.

### 1.1. Classification of Urinary Catheters [7]

The inclusion of medicinal substances in medical device development is gaining a growing popularity. Coated catheters are considered a combination of medical devices and medicinal products that provide additional benefits for patients. The pharmacological action of the medical substance is ancillary to the principal intended action of the device, which is achieved by physical or mechanical means, e.g., reusable inhalers, transdermal patches, medicated condoms, and drug-eluting stents. The new European regulation on medical devices was introduced on 26 May 2021 that stipulates more extensive requirements and classification rules [8]. According to this, urinary catheters are classified under Rule 5 as:

1. Class I, if they are intended for **transient use** (intended for continuous use for less than 60 min);
2. Class IIa, if they are intended for **short-term** use (intended for continuous use for between 60 min and 30 days) and;
3. Class IIb, if they are intended for **long-term** use (intended for continuous use for more than 30 days).

### 1.2. Types of Urinary Catheters

Urinary catheters can be external, urethral (including indwelling or intermittent), or suprapubic. Another factor for categorization is the catheter insertion tip and length.

Urethral catheters are invasive because they are inserted transurethrally.

#### 1.2.1. Indwelling Catheters/Foley Catheters

**Figure 1:**
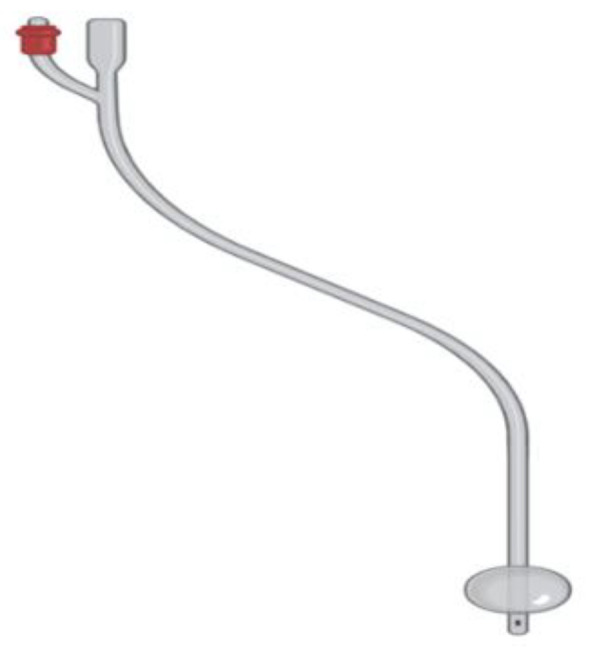
Indwelling catheter. (This figure is created by bio render [11]).

This is the most common type of catheter and is placed inside the bladder, either for a short or long period. If it is being used for a longer duration, then it should be changed every 3 months. The balloon on the catheter is filled with water, and one end of the catheter is kept inside the bladder and the other end drains the urine into the urinary bag [9,10]. In Figure 1 is presented indwelling catheter, used for emptying the bladder of the patient.

#### 1.2.2. Intermittent Catheters

**Figure 2:**
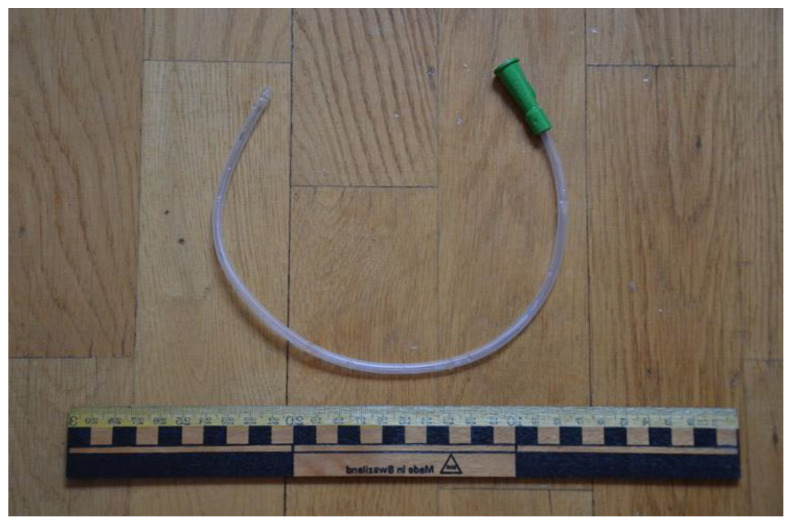
Intermittent catheter. Reprinted from ref. [13] under a creative common license (CC BY-SA 3.0), Originally created by Bengt Oberger.

These types of catheters are used multiple times (4–5) in a day for emptying the bladder whenever it gets full. It is inserted into the bladder through the urethra. The one end of the catheter is either left open-ended or attached to a urine bag to collect urine. When urine stops flowing through the catheter, it is removed [12]. In Figure 2 is presented intermittent catheter, which can be used multiple times a day by the user.

#### 1.2.3. Suprapubic Catheters

**Figure 3:**
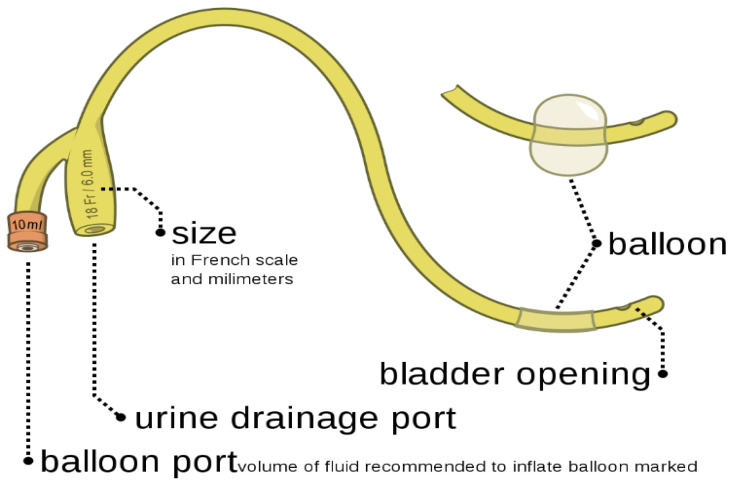
Suprapubic catheter. Reprinted from ref. [14] under a creative common license (CC BY-SA 3.0), Originally created by Olek Remesz.

These are the most invasive catheters as they require surgical procedures. The catheter is left in place in the bladder by making a small incision in the abdomen and placing it directly in the bladder, passing through the abdomen. The suprapubic catheters are used when the patient’s urethra is damaged, or they are unable to use an intermittent catheter. The chances of infection are low with these catheters [11]. Figure 3 represents the suprapubic catheters, can be used by performing surgical procedures.

#### 1.2.4. Condom Catheters

**Figure 4:**
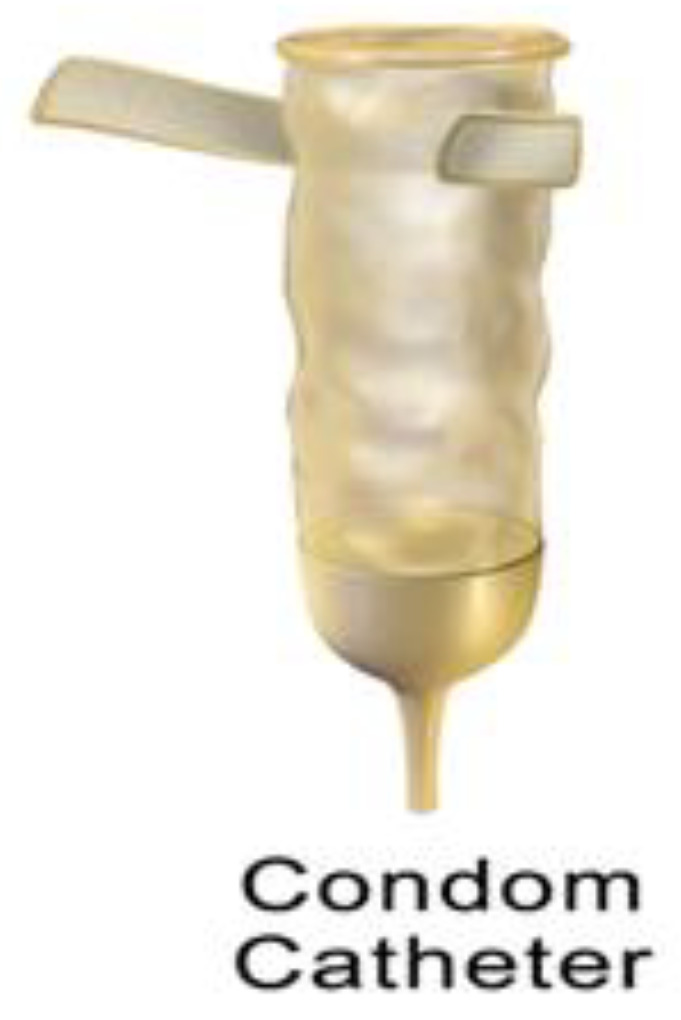
Condom catheter. Reprinted from ref. [16], under a creative common license (CC BY-SA 3.0), Originally created by Bruce Blaus.

Condom catheters are used by male patients suffering from urinary incontinence. A condom-like device is placed over the penis, and a tube from this device leads to the drainage bag. This type of catheter does not go inside the bladder and is only worn on the genital organ; to avoid the risk of infection, it should be changed every day [15]. Figure 4 represents the condom catheter, which can be worn over the genital organ by the user.

Most diameters of catheters range between 10 French to 20 Fr in size. The three main lengths of the catheters are: (a) male catheter length—16 inches; (b) female catheter length—6–8 inches; and (c) paediatric catheter length—10 inches [17,18].

Additionally, there are two main kinds of catheter insertion tips [19]: (a) **straight tip**—this is a standard tip of most of the catheters; and (b) **coudé** **tip**—coudé tip catheters are used by patients who have difficulty passing a catheter through their genital organ. Table 1 describes the different types of catheter coatings, materials and sizes currently available in the market for clinical use for the targeted population.

## 2. Urinary Incontinence

Urinary incontinence is a condition in which the patient loses control over their bladder, which is a very common problem affecting millions of people. It frequently occurs in elderly patients or might be associated with unhealthy food habits, constipation, an enlarged prostate, prostate cancer, a neurological disorder, a tumour in the urinary tract, menopause, or a urinary tract infection on a larger scale. Urinary incontinence can also lead to further complications, discussed below [21,22].

### 2.1. Vesicoureteral Reflux Caused by Urinary Incontinence

Normally, urine flows in one direction, travelling through the kidneys to tubes called ureters, where it gets stored in the bladder. When the bladder is unable to empty itself, it pushes the backflow of urine into the ureters and kidneys. This backflow of urine can cause infections and damage to the kidneys, such as the formation of stones in the kidneys [23,24].

The pathophysiological mechanism of urolithiasis in patients suffering from vesicoureteral reflux is related to urinary tract infections and urinary statis, which can further promote the formation of urinary crystals [25]. Normally, these stones can pass through the urine, but if they become large, they can cause a blockage in the kidney drainage system resulting in severe pain, bleeding, infection of the kidney, or failure [26].

### 2.2. Bladder Stones

**Figure 5:**
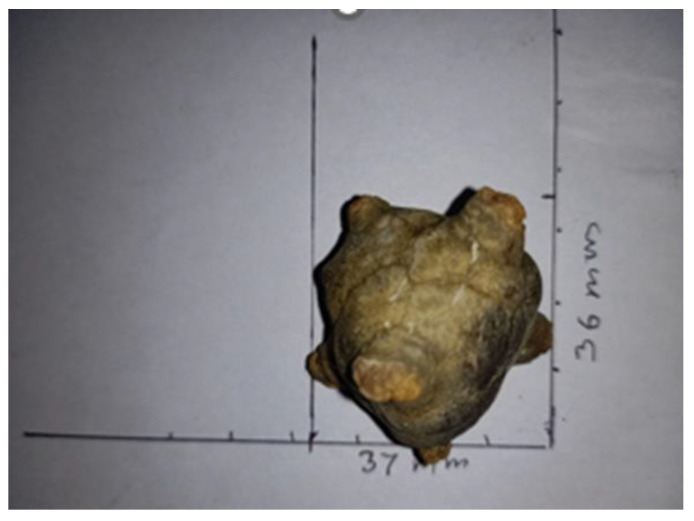
Bladder stones. Reprinted from ref. [32], under creative common license (CC BY-SA 4.0), Originally created by Vijayanrajapuram.

Urinary incontinence is the loss of bladder control, which includes the bladder’s loss of ability to empty itself. As a consequence of this, the leftover urine becomes concentrated, and the liquid starts to turn into crystals, forming bladder stones. The most common type of bladder stones is composed of uric acid due to the low urinary pH level inside the bladder. It promotes urate conversion to uric acid, which is less soluble and, therefore, makes it more prone to form crystals [27]. In a few cases, the stones are also composed of calcium oxalate, calcium phosphate, ammonium urate, cysteine, or magnesium ammonium phosphate (in the case of infection) [28,29]. Stones which are composed of calcium oxalate usually originate from the kidney. These stones can also pass-through urine when they are very small, but can also get stuck on the walls of the bladder or ureter, causing damage and blockage to the organs. These bladder stones can further cause discomfort or pain in the genital organ of males, irregular urination, pain in the lower stomach area, blood in urine, puss in urine, cloudy urine, chronic bladder dysfunction, or repeated urinary tract infections [30,31]. Figure 5 represents bladder stones, formed by the crystallisation of the concentrated leftover urine in the urinary bladder.

### 2.3. High Blood Pressure

Due to bladder incontinence, the bladder gets full frequently and this results in constant pressure on the kidneys, which leads to high blood pressure problems. Continuous high blood pressure then damages the arteries, making them less elastic and resulting in decreased blood flow and oxygen to the heart, causing chest pain and angina [33,34].

### 2.4. Urinary Tract Infection

Urinary incontinence is also a predisposing factor for urinary tract infections. The residual urine fosters bacteria growth, leading to Urinary Tract Infections (UTIs). Generally, catheterization poses a high risk for developing UTIs because bacteria can pass freely if the catheter is not cleaned or changed [35].

## 3. Complications Associated with Catheterization

### 3.1. Bacteriuria

Bacteriuria, also known as bacteria that is present in the urine, often occurs 2–10 days after insertion of the catheter. At the time of catheter insertion, numerous organisms are presented in the periurethral area which can be introduced into the bladder. Other reasons responsible for causing bacteriuria are: (1) the presence of remaining urine in the bladder because of inadequate bladder drainage, which can promote the growth of bacteria; (2) damage to bladder mucosa; (3) irritation caused by the usage of a catheter; and (4) biofilm formation on the surface of the catheter.

According to the literature, the duration of the catheterization process is highlighted as the most important risk factor for the development of bacteriuria and infection, and the infection risk increases by an estimated 5–10% per catheterization day [36]. Under short-term catheterization, 10–30% of the patients are likely to develop bacteriuria, whereas in the long-term catheterization process, the percentage number can increase to 90–100% of patients [37].

To prevent the incidence of bacteriuria, a closed system should be used to perform the catheterization process and the catheters should be removed as soon as possible after draining the bladder. Sealed drainage systems are also to be used to prevent bacteriuria. Catheter-associated bacteriuria is usually asymptomatic in nature and can be resolved normally once the catheter is removed [38].

### 3.2. Catheter-Associated Biofilms

A biofilm is a process of the collection of micro-organisms colonizing on the surface of a medical device. Once the catheter is inserted, bacteria quickly start developing colonies on the surface of the catheter known as biofilms, which can stick to the catheter surface and drainage bag. Urine proteins play a key role in the initial process of biofilm formation [39]. These biofilms can be composed of single organisms, but can also lead to multiorganisms if the catheter is exposed for a longer period of time [40].

Biofilms and infections are linked as biofilms provide a reservoir for micro-organisms, which leads to the production of urease. As urease is produced, the urine becomes alkaline, leading to the production of ammonium ions, followed by the crystallization of magnesium phosphate and calcium in the urine. These crystals then become attached to the biofilms, resulting in an encrustation of the catheter [41].

Biofilms can start to develop within 24 h of the catheter insertion. The biofilms provide a protective environment for the micro-organisms, which prevents the effect of antimicrobial agents.

### 3.3. Encrustations

Once the minerals get deposited with the biofilms on the surface of the catheter, they result in encrustations. These can be found on the inner side of the catheters and can block the catheter tube to stop the flow completely. They may also make a coating around the balloon, making it hard to deflate. Encrustation on the catheter occurs in the long-term catheterization process. A raised urinary pH as a result of infection with urease-producing bacteria is a key element in the encrustation process; however, encrustation can take place when there is a lack of infection as well [39]. Patients who have an alkaline pH usually are more prone to catheter encrustations, as they are directly related.

### 3.4. Urosepsis

Urosepsis is an untreated urinary tract infection that spreads to a person’s kidney and can become life-threatening.

### 3.5. Urethral Damage

Male patients with orchitis, a scrotal abscess, prostatitis, and epididymitis are likely to develop urethral damage when the catheter is inserted, and the risk increases with long-term catheter use. If difficulty in passing the catheter has been encountered, it means that the urethra has been damaged by creating a false passage into it or the passage is blocked by the prostate, bladder neck, or sphincter [42].

Urethral complications include the following conditions: (a) tearing of the urethra; (b) developing urethral fistulas; (c) inflammation in the urethral meatus; (d) bladder inflammation by scrotal abscesses; (e) epididymitis; (f) bladder stones; (g) bladder cancer; and (h) catheter expulsion [43].

### 3.6. CAUTI (Catheter-Associated Urinary Tract Infection)

Catheter-related problems persist as long as a person is using urinary catheters. In most cases, use of catheters leads to a healthcare-associated urinary tract infection [44].

CAUTIs are the most common infection which is acquired in hospitals or nursing homes and comprises more than 40% of nosocomial infections. CAUTIs are associated with the long-term catheterization process and are defined as complicated urinary tract infections. Patients who are using indwelling catheters are prone to develop an infection, with CAUTIs at least two times a year and, also, require hospitalization [44].

CAUTIs can further deteriorate into urosepsis (untreated UTI spreading to the kidney) and septicaemia (blood infection). The usage of catheters makes a patient prone to different kinds of infections, as catheters can impregnate the organisms leading into the bladder and can help in bacterial adhesion by promoting colonization, which can further cause mucosal irritation. CAUTIs involve various organisms and bacteria ranging from *E. coli*, *Pseudomonas aeruginosa*, *enterococci*, *staphylococci*, and *Candida*. Women are more likely to be affected by CAUTIs as compared to men because of the proximity of the urethra to the anus [45].

Most bacteria responsible for CAUTIs enter the urinary tract through catheters either extraluminally or intraluminally and can cause bacteriuria. Extraluminal entry of bacteria is most common as it occurs when the inserted catheter becomes contaminated by any source or micro-organisms, which move from the perineum to the surface of the catheter. In most cases, the faecal strains contaminate the perineum and the urethral meatus, and then, move along the external surface to the bladder to cause bacteriuria. Intraluminal contamination occurs when a bacterium is transmitted from a contaminated catheter, drainage bag, or tube. The migration process of bacteria from the catheter to the bladder takes up to 1–3 days [46].

Different antibiotics are used to treat UTIs, but due to their growing use now, there has been an increase in antibiotic-resistant micro-organisms, especially among *P. Aeruginosa* and *C. albicans*. For patients undergoing long-term indwelling catheterization, the symptoms of UTIs are nonspecific. These symptoms occur when the epithelium of the urinary tract gives an inflammatory response to the colonization of bacteria. Patients may experience symptoms such as pain and fever [47].

## 4. Prevention and Management of CAUTIs

Catheter-associated urinary tract infections are very common in patients undergoing the catheterization process. The associated encrustation is also affected by catheter surface properties such as roughness, hydrophobicity, wettability, charge, polymer chemistry, and coatings. In spite of this, different materials and coatings for catheters have been developed, but an ideal material for manufacturing or coating, especially for long-term catheterization, is still under research [3,48,49].

Modifications Offered in The Urinary Catheter

The two types of modifications which are offered for the prevention and management of CAUTIs are mentioned below:

- Modifying the material used for production of catheters, such as latex, polyvinyl chloride (PVC), siliconized latex, silicone elastomers, polytetrafluoroethylene (PTFE)-coated latex [50,51], hydrogel-coated latex [52,53], enzymes, polyethylene glycol [54,55], and polyzwitterion coatings [56,57];
- Applying the antimicrobial coating on the surface of urinary catheters, such as metal ions, antibiotics, nitric oxide, antimicrobial peptides, and bacteriophages.

### 4.1. Approaches to Prevent CAUTIs by Modifying Materials

#### 4.1.1. Hydrogels

This is a group of swellable, insoluble, and hydrophilic polymers. Hydrogels show solid-like properties, an ideal property for catheter material. Hydrogels can be coated by either using natural polymers such as gelatin and chitosan, or synthetic polymers such as poly(vinyl alcohol), poly(ethylene glycol), and poly(sulphobetaine). The simplest technique of coating a hydrogel on the catheter surface is achieved by dip coating. However, the interaction between the coating and surface can have a weak, noncovalent force and can result in delamination of the coating. It is a challenge to increase the adhesive properties of hydrogel coatings. Changwen Zhao et al. recently investigated a wide range of techniques to achieve anchoring a hydrogel layer on the surface, e.g., anchoring hydrogel layers by click chemistry, anchoring hydrogel layers by free-radical polymerization, anchoring hydrogel layers by photochemical coupling, anchoring hydrogel layers by dopamine-functionalized polymers, and anchoring hydrogel layers by self-condensation of silane [58]. As a coating, hydrogels swell up and form a hydration layer, which will increase the hydrophilicity and which will act as a barrier for preventing protein adsorption [59]. According to the literature, the hydrophilicity of the coating pertains to antifouling properties, but in practice, the results vary. Clinical trial studies have been carried out that compared different types of catheters such as (a) siliconized latex, (b) pure silicone, and (c) hydrogel-coated silicone, and the results have showed that usage of silicone catheters results in low urethral inflammation and also resists encrustation, whereas usage of the hydrogel-coated catheter successfully reduced the formation of encrustation on the catheter surface [52,60]. In other studies, it was found that the hydrogel catheters were responsible for catheter blockage [56].

#### 4.1.2. Poly(Tetrafluoroethylene) (PTFE) Coating

PTFE is also known as Teflon, and it is also commercially available. PTFE coatings have excellent non-stick properties and can resist bacterial colonization [50]. This coating is a hydrophobic material and this material does not have swelling characteristics; therefore, it does not increase the catheter size in the body.

Few clinical trials have been conducted, and the data have been compared for the difference in blockages in: (a) silicone catheters available commercially, (b) silicone-coated catheters, (c) Teflon-coated catheters, and (d) latex catheter. All of these were observed for two weeks and the results showed that the silicone catheter had a lower rate of blockage in comparison to latex- or Teflon-coated catheters [61]. Another study compared the Teflon-coated catheter with the silicone- and hydrogel-coated catheters for their potential to prevent bacterial colonization and reduce mucosal irritation, and the results of the study showed that the hydrogel-coated catheters have better properties in comparison with Teflon-coated or silicone catheters [62].

#### 4.1.3. Polyethylene Glycol (PEG Coatings)

Polyethylene coatings have properties such as being nonantigenic, nonimmunogenic, and protein repellent [54]. PEG can also have antifouling properties in combination with antibacterial cations coated on silicone and a polycarbonate brush containing dopamine. The polymeric brush layer of PEG blocks the interaction between the substrates and the bacteria and can result in a repulsive steric effect [63]. An in vitro study shows that PEG can block bacterial adhesion. The study focused on the strains of *E. coli* and *Staphylococcus epidermidis* using lab-defined media, and it was found that PEG prevented the adhesion of *E. coli* on the polyurethane surface, but is not successful against *Staphylococcus epidermidis* [50]. PEG appears to be a good antifouling material for in vivo studies as it can also be used for permeating chlorhexidine into catheters, which is a well-known antiseptic and disinfectant [64,65].

#### 4.1.4. Polyzwitterions

Polyzwitterions use their property of low surface energy, which results in repulsion. The mechanism of action behind these polymers is that they have both positive and negative charge, resulting in a neutrally charged molecule, which further results in a hydrophilic layer responsible for repelling the binding of bacteria [66]. Polyzwitterions are strongly hydrated under physiological conditions and, thus, result in reduced adhesion of biological matter (antifouling properties) on the surface of the material. They also have protein repellence and cell compatibility properties, making them a promising coating for catheters, implants, and wound dressings [67,68]. An in vitro study of these polymers has observed and evaluated their capability of repelling *P. mirabilis* colonization, and the results have shown that this modification can neither repel nor prevent *P. mirabilis* colonization, biofilm formation, or encrustation of the catheter on either silicone- or latex-based catheter [69]. The poly (sulfobetaine methacrylate) (PSBMA)-modified section showed a reduction in biofilm formation from *P. Aeruginosa* and *S. aureus*. Though studies have been performed, additional research is still required to characterize the antifouling activity in reducing microbial colonization [57,70,71].

#### 4.1.5. Enzymes

The role of enzymes for antibiotic activity includes quorum quenching, hydrolytic bonds, and reversible bonds. These few enzymes working against bacterial catheter colonization are discussed below:

(a) **Acyclase**—Acyclase, in combination with alpha-amylase, was tested for quorum quenching. Quorum quenching activity is responsible for inhibiting biofilm formation, which is why the risk of developing drug resistance is minimal in this case [53]. An in vitro study has been carried out with the coating of acylase and alpha-amylase, and the results show that the coating was successful in significantly reducing the biofilm formation by *P. aeruginosa* by 40%, and by *S. aureus* by 30%. In in vivo studies, the biofilm formation was decreased by 90% on the catheter balloon section [72].
(b) ∝−chymotrypsin**(**∝−CT**)**—∝−CT is a serine endopeptidase which attacks the unreactive carbonyl group and breaks the peptide bonds [73]. Based on this property of ∝−CT, its ability to disrupt biofilm formation has been studied in an in vitro study as the matrix includes proteins, polysaccharides, and extracellular DNA [74,75]. In the in vitro study, ∝−CT was immobilized on a low-density polyethylene and nurtured with LB media containing *E. coli* MG1655 in a CDC biofilm reactor performing continuous stirring. The study showed a reduction in the no. of adherent cells, and in biofilm thickness, roughness, and coverage [75].
(c) **Exopolysaccharide-specific Glycoside hydrolase**—Exopolysaccharides are a major component of bacterial biofilm development, resulting in protection against antibacterial agents [74]. Glycoside hydrolases target and hydrolyse the glycosidic bonds of exopolysaccharide components of the biofilm matrix. Glycosides have properties of anti-biofilm agents [76,77]. Baker and collaborators investigated the glycoside hydrolase activity in a treatment against Pseudomonas aeruginosa biofilm development [78]. *P. aeruginosa* cultures were diluted in Luria–Bertani media (LB), and then, these diluted cultures were added to sterile 96-well polystyrene microtiter plates and nurtured for 24 h under controlled conditions. Glycoside hydrolase was added in different concentrations at different times at 0, or in developed biofilm conditions. The results show a significant reduction and disruption in biofilms [76].

In another study, a specific ***Ghs*** was used which targets ***Psl*** (polysaccharide synthesis locus), a neutral exopolysaccharide, and immobilized it to silica glass, PDMS, and polystyrene surfaces. *P. aeruginosa* was grown on the ***PslGh***- immobilized surfaces at different points of time for up to a week, and they found that ***PslGhs*** were able to reduce three times in the surface-associated bacteria [78]. In conclusion, only hydrogel- and PTFE-coated catheters studied in clinical trials have shown contradictory results from all the antifouling surfaces. Despite these contradictory results on hydrogels catheters, their commercial demand and availability have made them popular [52,53]. Hydrogel catheters are commonly coated and used in hospital settings also, but the evidence of their efficacy is still uncertain [52].

## 5. Approaches to Prevent CAUTIs by Antimicrobial Coatings

**Figure 6:**
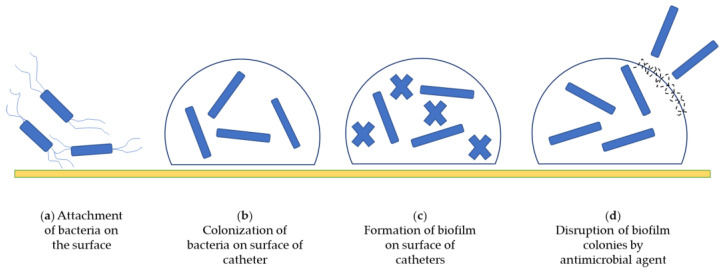
Disruption of biofilm colonization on surface of urinary catheter by antimicrobial action.

Antimicrobial functions can be achieved by targeting microbes in different ways to inhibit colonization on the surface of catheters, such as: (a) by modifying the surface of catheters to prevent microbial adherence; (b) by contact killing; (c) by biofilm disruption, (d) by inhibiting unique metabolic steps; and (e) by releasing antimicrobial agents [6]. Figure 6 describes the life-cycle process of attachment of bacteria on catheter surface resulting in formation of biofilms and the disruption of biofilm by use of an antimicrobial agent.

**Figure 7:**
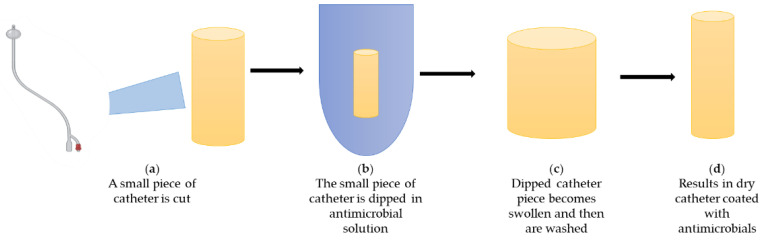
Description of incorporation of antimicrobial coating on urinary catheters.

Due to all of these properties, antimicrobial agents tend to be the most popular coating. The most popular antimicrobials which can be used for coatings are antibiotics, metal ions, nanoparticles, nitric oxide, antimicrobial peptides, bacteriophages, and natural bioactive molecules. Figure 7 describes the process of incorporating antimicrobial agents on urinary catheters.

### 5.1. Antibiotics

Antibiotics are a recommended treatment for CAUTIs, as they are used for fighting bacterial infections. Once a urinary catheter is placed for a longer period of time (days or weeks) inside the bladder, then it can increase the risk for developing infection in the bladder or in the urethra. To avoid the spread of infection and to eliminate pathogens, the administration of antibiotics is required either locally or systematically. Catheters coated with drug-containing formulations act as drug delivery systems and are placed inside the bladder through the urethra to inhibit the bacterial infection inside. A few examples of devices which are used as drug delivery systems are: (1) the UROS infuser—it is a drug reservoir filled with a drug solution that has a pressure-responsive valve; (2) an intravesical balloon—it is inflated with drug formulations and is positioned inside the bladder; and (3) silicone tubes— they are prefilled with drug formulations [79]. Research has been carried out on antibiotic-coated urinary catheters for use over many years. However, due to the inconsistent results from in vitro studies and the high cost of clinical trials, their efficacy remains under question. The most common antibiotics which have been studied are nitrofurazone, gentamicin, triclosan, chlorhexidine, and fluoroquinolones (ciprofloxacin, sparfloxacin, and norfloxacin) [80,81,82].

#### 5.1.1. Nitrofurazone

Catheters are available as antimicrobial catheters in clinics. They inhibit the replication of DNA, resulting in inhibiting the growth and formation of biofilm [83]. Nitrofurazone-coated catheters have been tested against *S. aureus*, *E. coli*, *K. pneumoniae*, and *Enterococcus faecium*, and growth of the strains was found with the exception of *Enterococcus faecium*. Although this study has shown promising results, the nitrofurazone-impregnated catheters have shown mixed results in hospital settings. A clinical trial study was performed comparing nitrofurazone- impregnated silicone catheters and simple, noncoated silicone catheters and it was found that there is no effect on CAUTIs or bacteriuria. In another clinical trial, the nitrofurazone-impregnated catheters were compared with the uncoated catheters by testing them on the patients catheterized for less than 1 week, and it was found that there is a decreased rate of bacteriuria in the nitrofurazone-coated catheters [84]. When testing for new material coatings, a patient’s comfort is also an important factor to take into consideration. Nitrofurazone catheters have tended to be uncomfortable for use in patients. In May 2002, USFDA banned the use of nitrofurazone as a topical antiseptic for the treatment of animals and humans as it was found to be carcinogenic. As this ban was applied to nitrofurazone for over-the-counter use, catheters are not included under this; still, the interest in research on nitrofurazone catheters has slowed and has turned towards other antibiotics [3,52].

#### 5.1.2. Gentamicin

Gentamicin belongs to the broad-spectrum aminoglycoside antibiotics. Its mechanism of action acts by inhibiting protein synthesis through binding to the 30S ribosomal subunit of bacteria. A study has been performed in a rabbit model of CAUTIs and gentamicin-coated catheters were tested, and the results showed a significant reduction in incidence and severity of infection in the short-term catheterization process [85,86,87]. In another study, gentamicin also showed sustained release effects when PEG was added, extending the release profile for 12 days. This study focused on the release of gentamicin and found out that the release of gentamicin can be manipulated by varying the concentration of PEG and gentamicin initially. Although many in vitro and in vivo studies have been carried out, there is not enough supporting evidence in place to describe the use of gentamicin-coated catheters in clinical trials unfortunately [88].

#### 5.1.3. Norfloxacin

Norfloxacin has been tested in in vitro studies against *E. coli*, *Pneumonia*, *P. vulgaris*, and *P. aeruginosa*, and it has showed a significant reduction in the growth of these bacteria [89,90]. Norfloxacin has shown better efficacy and a sustained release profile when combined with other antibiotics (such as ciprofloxacin and azithromycin) against biofilm formation and preventing microbial growth. Many studies have shown that norfloxacin-coated catheters have induced a reduction in bacterial growth and effective bactericidal activity; still, their efficiency in vivo and in clinical trials has not been evaluated [91].

#### 5.1.4. Ciprofloxacin

Ciprofloxacin is an antibiotic which functions as a nucleic acid synthesis inhibitor and belongs to the fluoroquinolone category. It is a broad-spectrum, second-generation antibacterial agent. Ciprofloxacin mostly works against Gram-negative bacterial infections, skin infections, ophthalmic infections, bone infections, and respiratory and urinary tract infections [92]. Ciprofloxacin-coated catheters have been tested in rabbit models in comparison with uncoated hydrogel catheters against *E. coli* for CAUTIs, and it was found that the incidence of CAUTIs was delayed in the ciprofloxacin-coated catheters compared to uncoated catheters [93].

#### 5.1.5. Sparfloxacin

Sparfloxacin-coated latex catheters were tested against *E. coli* and *S. Aureus* in comparison with silver-coated and uncoated catheters using both broth and agar diffusion assays, and the results showed a reduction in the growth and binding of bacteria in the sparfloxacin-coated catheters in comparison to others. Aside from this study, no other in vitro and in vivo studies have been reported using sparfloxacin-coated catheters [94].

#### 5.1.6. Triclosan

Triclosan works by inhibiting the enzymatic activity responsible for fatty acid synthesis, as fatty acid is responsible for phospholipid formation and, thus, membrane formation [95]. At low levels, it has bacteriostatic activity and at high levels, it shows bactericidal activity [96]. Triclosan has been used in household products such as soaps and toothpastes as an antiseptic for decades, and recently, it has also been proposed for use as an antimicrobial coating for catheters, as it possesses good chemical properties, making it easy to coat on a catheter surface [97,98]. Although many in vitro studies have been carried out, its effect on the human body is still in question and the usage of triclosan in hospital facilities and other healthcare settings has been stopped due to safety concerns. Recently, it has also been found that triclosan develops resistance to bacteria both in vivo and in vitro, which adds to the controversy related to use of triclosan [99].

#### 5.1.7. Chlorhexidine

Chlorhexidine acts differently on both bacteria and fungi. Due to its low toxicity in mammals, it is commonly used for meatal cleaning before placing the urinary catheter [65]. Chlorhexidine at low levels has bacteriostatic activity, and at high levels it has bactericidal activity. Coating catheters with chlorhexidine has been performed by different techniques, such as spray coating, dip coating, etc. [100]. Chlorhexidine has been coated in the form of nanoparticles and varnishes, and the results showed a decrease in the adhesion of bacteria, which results in preventing biofilm formation [101,102]. Chlorhexidine also shows sustained release properties if conjugated with some compatible polymer. When compared with other antibiotics, it is effective for a longer period without developing bacterial resistance, and also, the reports of reactions with chlorhexidine are very rare. Once enough data from the study of chlorhexidine-coated catheters is generated, this coating can be very patient friendly and will be in demand [103,104,105].

### 5.2. Metal-Based Approaches

#### 5.2.1. Silver Ions

These are the most common clinically tested coatings available for urinary catheters. Studies have been carried out in in vitro animal models and in clinical trials, and the variation in results from the studies puts a question on the effectiveness of the coatings [50,106,107,108,109]. Silver ions act by disrupting the membrane proteins, resulting in oxidative stress by releasing silver ions in the bladder [110,111]. An in vitro study has been conducted comparing a silver silicone-hydrogel catheter, nitrofurazone-coated silicone catheter, silicone-hydrogel catheter, pure silicone catheter, silver-latex-hydrogel catheter, and latex-hydrogel catheter against *E. coli* and *Faecalis*. Catheter pieces were cut and incubated in sterile media for a few days, and then the coatings were tested against the bacterial strains. It was found that there is no effect on bacterial binding in silver-coated catheters, and nitrofurazone-coated catheters showed a lower rate of bacterial binding. As per the results of this study, the silver urinary catheters have no effect on the prevention of CAUTIs or bacterial adherence [112,113].

Another study compared three different catheters, including silver-coated catheters, sparfloxacin-coated catheters, and uncoated-siliconized latex catheters, against the bacterial strain of *E. coli* and *S. epidermidis* for 3 days. The results showed that the sparfloxacin-coated catheters have a lower rate of bacterial adhesion in comparison to the silver-coated and uncoated catheters [94].

In vivo studies have also been performed on mouse models against *E. coli*. The silver-coated silicone catheters were placed in the bladder, followed by the bacterial strains [71]. After two weeks, the catheters were studied for the overall colonization of bacteria, and it was found that the coated catheters showed reduced colonization of bacteria as compared to the uncoated catheters [114].

In clinical trial studies, the silver-coated catheters have shown different results. There are many studies which suggest that silver-coated catheters can reduce the occurrence of CAUTIs as compared to the uncoated catheters [115].

A study compared silver-alloy hydrogel catheters with the hospital’s standard catheter, and the results showed that silver-alloy hydrogel catheters reduced the occurrence of CAUTIs up to 60%. On the other hand, some studies have shown no advantage of using silver-coated catheters [116,117].

Analyses of cost have also been carried out for different modified catheters by some review articles, and it was concluded that it is very unlikely for silver-coated catheters to be cost-effective as compared to nitrofurazone-coated or other coated catheters [52,62].

Overall, diverse results have been shown by silver-coated catheters, and also, they are not cost effective, which makes it difficult to justify their use. As per the evaluation of the research, further analyses are needed for evaluating the effectiveness of silver coatings.

#### 5.2.2. Nanoparticles

Nanoparticles are an ideal choice for the drug delivery system as they improve bioavailability by improving the stability and solubility of the drug. Nanoparticles are used to deliver metals such as silver, gold, copper, etc. [118].

**Silver nanoparticles**—silver nanoparticles have the property of binding to proteins or molecules irreversibly, resulting in decreased microbial activity. Studies have shown that silver-coated nanoparticles reduce the incidence of biofilm formation and also prevent growth of several pathogens such as *E. coli*, *Enterococcus* spp., *S. Aureus*, *P. aeruginosa*, *staphylococcus*, and *Candida albicans* [119]. In the studies, it was found that the silver nanoparticles do not induce any inflammation or toxicity in the body and the molecules are easily removed from the body through the faeces in a period of 10 days. Although the silver nanoparticles have shown positive results, their antimicrobial property is still in question [120].

**Gold nanoparticles**—Gold nanoparticles have a mechanism of action based on becoming attached to the membrane of the bacteria, which is followed by disrupting the potential of the membrane and reducing the ATP levels, as well as inhibiting the transfer of tRNA binding on the ribosome. These nanoparticles have been tested against different bacteria such as *S. aureus*, *K. pneumonia*, *P. aeruginosa*, and *Enterococcus faecalis*. Studies have been performed to assess the efficacy of gold nanoparticles, and not much bacterial inhibition was documented till 48 h, which brings the efficacy of gold nanoparticles into question [121].

**Copper nanoparticles**—Copper shows its antimicrobial properties by affecting the bacterial cell when entering into DNA, binding to its phosphate site, and thus, degrading DNA, inactivating the essential bacterial enzymes and causing membranes and cell wall disruption [122,123,124,125]. Studies have been performed for copper in combination with silver on the surface of a polyurethane catheter against the bacterial strain of *E. coli*. [126]. The coated and uncoated catheter samples were taken and were exposed to the bacterial strain, and then checked for traces of colonization of bacteria on the catheter surface. The results showed that by 2 min of interaction between the strain and the surface of the coated catheters, no formation of colonies was detected [127].

However, all the metal-based coatings have shown mixed results, and only silver ions and nanoparticles have been tested on a larger scale. The most important to study is the host factor which is responsible for efficacy. Studies have also shown that copper in combination with zinc has shown better antimicrobial activity, but we still need to research the toxicity levels of these coatings before they come into practice.

### 5.3. Nitric Oxide (NO)

Nitric oxide covalently binds to DNA, lipids, and proteins and further inhibits or kills the pathogens. It has been used for many years as an antimicrobial in biomedical applications [128,129]. A study has shown that in in vitro models, the *NO* is impregnated in a gaseous form into the catheter, and then it shows sustained release of *NO* for two weeks. Nitric oxide has also shown results for preventing biofilm formation and the growth of pathogens. Although NO has shown promising results in treating vasodilation, angiogenesis, wound healing, and neurotransmission, its effects in the bladder are still needed to be further studied in depth [128].

### 5.4. Bacteriophages

Bacteriophages can be a good coating for catheters as they are viruses specific to bacteria and possess certain properties, such as:

(a) They can act as biofilm-controlling agents because of their property to target specific pathogens.
(b) They have self-replicating properties in the presence of their host cells.
(c) They are used effectively against the bacteria, which are multidrug resistant.
(d) If multiple phages are combined, they show better results in the treatment.

Numerous studies have been performed on bacteriophages, which gives evidence of their property to inhibit or prevent biofilm formation. Based on their property to delay biofilm formation, they can be used for short-term catheterization [130]. Several studies have been carried out and the effectiveness of the catheter coated with bacteriophages has been tested against the bacterial strains of *P. mirabilis*, which is mainly responsible for biofilm formation; *P. aeruginosa*; *E. coli*; and *S. epidermidis* for catheter colonization. An in vitro study was performed, in which phage 456 was coated onto a hydrogel Foley catheter and was tested against the bacterial strain of *S. epidermidis* for biofilm formation. The catheter was exposed for a 24 h period and the results showed that there was a subsequent reduction in biofilm formation [131]. In another study, hydrogel-coated Foley catheters were coated with bacteriophages and were tested against the strains of *E. coli* and *P. mirabilis*. The catheters were exposed to the strains for 24 h. This study showed that bacteriophages were able to prevent and minimize the formation of biofilm up to 90% [132].

In another in vitro study, the strains of *P. aeruginosa* and *P. mirabilis* were used by incubating the bacteriophage-impregnated catheter into the solution of phages for 1 h. The catheters were monitored for 3–4 days for biofilm formation, and it was found that the biofilm formation was minimized by four times with *P. aeruginosa* and two times with *P. mirabilis* [130].

Moreover, many studies have been performed which prove that multiple phage coatings show more effectiveness than single phages. In conclusion, catheters coated with bacteriophages have potential as an antimicrobial catheter as they are species-specific and do not promote antimicrobial resistance.

### 5.5. Antimicrobial Peptides (AMPs)

Antimicrobial peptides show antimicrobial activity against different pathogens [133]. *AMPs* destabilize and permeate the cell components and bacterial membrane [134]. The sources of *AMPs* are humans, plants, bacteria, and animals, and they can be synthesized in the lab also [135]. The antimicrobial property of *AMPs* varies from peptide to peptide. Thus, we will review the antimicrobial activity of different *AMPs* [133].

An in vitro study has been completed on two-short *AMPs* rich with arginine, lysine, and tryptophan that were attached to an allyl glycidyl ether polymer brush and coated on silicone catheters, which was tested against the bacterial strains of *E. coli*, *S. aureus*, and *C. albicans*. It was found that these *AMPs* were successful in reducing the formation of biofilm formation for 3 days and also killed almost 80% of the bacterial strains [133].

Another study showed that the *AMP* chain 201D from crowberries also shows antimicrobial activity against *E. coli* and *S. aureus* when coated on a silicone catheter surface [135]. In another in vitro and in vivo study, polyurethane catheters were coated with synthesized cysteine-labelled peptide E6 by a polymer brush coating and were tested for CAUTIs. The results showed that there was a reduction in bacterial colonization of *P. aeruginosa* and *S. aureus* on surface catheters and the bladder [136]. Despite the fact that *AMP* research is in the early stages now, many of them show great potential; however, we need further studies in in vivo and clinical study models to obtain a better idea of the effect of AMPs on the bladder.

## 6. Authors’ Perspective

### 6.1. Ambiguities in Testing New Coatings

Tremendous efforts have been made to prepare a new antimicrobial coating for urinary catheters to prevent microbial colonization and biofilm formation, but the variations resulting from the in vivo, in vitro, and clinical trials make it difficult to give a specific evaluation of the coatings.

Most of the in vitro studies are using laboratory culture media instead of human urine samples to study the biofilm formation activity. Several studies have shown that laboratory culture media do not imitate the urinary bladder environment. Therefore, pathogens show different activity when placed in laboratory conditions [137,138,139].

Another factor which raises concern is the concentration of oxygen, which is required for bacterial growth in urine culture. Many in vitro tests are performed under shaking conditions, where oxygen is supplied into the system to accelerate bacterial growth. It has been noticed during the studies that the amount of oxygen concentration which is incorporated into the system is greater than the amount of oxygen concentration found in actual urine samples from patients and healthy individuals. This suggests that in vitro study conditions do not mimic the in vivo study conditions, and thus, results vary [140,141].

Another issue concerns the environment of the bladder. Various studies reported that when a catheter is used in an animal or human model, it causes trauma to the lining of the bladder, which further induces an inflammatory response of releasing serum proteins and changes the environment of the bladder [142,143,144]. These serum proteins, when released, accumulate on the surface of the catheter, and hence, modify the effect of the antimicrobial coating [142,144,145].

Other factors due to which studies show contradictory results include: (a) choosing the right type of assay for both an antifouling and antimicrobial coating; (b) the inhibitory concentration; and (c) the duration of experiments.

Due to a lot of variation in results, it is difficult to comment on the potential and efficacy of different coatings and materials.

### 6.2. 3D Printing and Regulatory Aspect

The incorporation of medical substances in medical devices is becoming more popular due to the emergence of 3D printing technology [8]. As a powerful and dynamic multifunction production method, 3D printing is growing beyond the constraints of traditional methods and delivering a prominent capacity for manufacturing tailored devices, including drug-eluting catheters [146]. According to the literature, fused deposition modelling (FDM) is the only adapted system for designing coated catheters with promising results. A filament of a thermoplastic polymer is used in 3D printing layers of materials with a fused deposition modelling (FDM) approach [147]. In addition, 3D printing is a low-cost method and the most important benefit of using this technology is the capacity for producing adjustable shapes with complex microstructures, in line with the demands of patients. In this way, drug doses can be easily adjusted by modifying the size and shape of the catheters. A coating of multiple drugs on catheters with different release profiles is also achievable by 3D printing [147]. Despite the advantages of 3D printing technology, there are various challenges, such as the achievement of aseptic production and selection of materials with constant exposure to radiation. In order to evaluate the outcome of the 3D printing technologies, it is necessary to review the stability, repeatability, and reproducibility aspect of the process. Even though the application of 3D printing in medical device development has significantly advanced, it still needs to be rigorously assessed to meet the standards on the grounds of efficiency, energy usage, and sustainability. Looking through the regulatory aspects, the rapidly growing 3D printing technology for medical devices needs to fit into the current regulatory regulations, as some of the devices do not fall into the current framework; therefore, some regulatory improvements are necessary. Currently, the major regulatory hurdles for 3D printing of catheters include monitoring the printing process, testing properties of final products, and implementing the patient-specific acceptance criteria [146].

## 7. Conclusions

Currently, research on antimicrobial coatings using a wide range of materials is booming. However, there are still some challenges that need to be addressed to improve the coatings in the future, or to create something new for urinary catheters. The major issue in the development of antimicrobial coatings is the presence of antibiotic resistance. The usage of antibiotic-coated catheters is going to be affected as antibiotic resistance continues to increase. In this case, multimechanism approaches can reduce the resistance issue. These approaches can be employed by just using only one antimicrobial agent or using synergistic approaches, such as by making the coating or material of the catheter with either one or two bactericidal agents and then top coating it with an antifouling agent. Another strategy is the use of nanoparticles in coatings. One of the challenges faced by antimicrobial coatings is cytotoxicity, which is induced either through direct contact with the antimicrobial agent or the leaching of the agent into the patient’s body. Another one is the local delivery of the antimicrobial agent at the time of infection. Finally, the patient factor is the prime concern which needs to be taken into account, especially for long-term catheters. In this case, a selection of catheter coatings which are well lubricated, flexible, and able to prevent blockages in the inner lumen are much preferred for the patient’s overall welfare. In conclusion, a comprehensive medical judgement is required on an individual basis to consider all the surrounding factors determined by the patients’ medical condition in line with the availability of the catheter coatings.

## Figures and Tables

**Table 1:** Currently available catheter types in clinical use [20].

Type of Coatings for Catheter	Materials for Catheter	Size Available in Fr	Target Population	Tip of Catheter Available
		(Paeds—6–10 Fr)		
		(Female—10–12 Fr)		
		(Male—14–18 Fr)		
		(Clot Retention—20–26 Fr)		
Hydrogel-coated catheters	Latex, polyvinyl chloride (PVC), red rubber, silicone	8,6,10,16,18	Paediatrics, males, and females	Straight and coudé tip
Silver-coated catheters	Latex, PVC, silicone	6,10,12,16,17	Paediatrics, Males, and Females	Straight and coudé tip
Hydrophilic-coated catheters	Silicone, vinyl, polyurethane, polyolefin-based elastomer (POBE), PVC, red rubber	5,6,8,10,12,14,16,18, 19,20,22	Paediatrics, Males, and Females	Straight and coudé tip
Pre-lubricated catheters	PVC, red rubber	12,14,16	Males and Females	Straight and coudé tip
Polytetrafluoroethylene (PTFE)-coated catheters	Latex	16	Males	Coudé tip
Uncoated catheters	Silicone, latex, PVC	6,8,10,12,14,16,18,22	Males and Females	Straight and coudé tip

## Data Availability

Not applicable.

## Abbreviations

indwelling urinary catheters (IDC), urinary tract infection (UTI), catheter-associated urinary tract infection (CAUTI), polytetrafluoroethylene (PTFE), polyethylene glycol (PEG), polyvinyl chloride (PVC), polysulfobetaine methacrylate (PSBMA), Luria–Bertani media (LB), polysaccharide synthesis locus (Psl), glycoside hydrolase (Ghs), United States Food and Drug Administration (USFDA), adenosine triphosphate (ATP), transfer ribonucleic acid (tRNA), deoxyribonucleic acid (DNA), nitric oxide (NO), antimicrobial peptides (AMPs), fused deposition modelling (FDM), *Pseudomonas aeruginosa* (P. aeruginosa), *Escherichia coli* (*E. coli*), *Candida albicans* (*C. albicans*), *Staphylococcus aureus* (*S. aureus*), *Klebsiella pneumoniae* (*K. pneumoniae*), *Proteus vulgaris* (*P. vulgaris*), *Staphylococcus epidermidis* (*S. epidermidis*)
